# Quantitative duplex ultrasound assessment of superficial venous incompetence: A state-of-the-art review

**DOI:** 10.1177/1358863X261419731

**Published:** 2026-03-23

**Authors:** Johan Skoog, Oskar Nelzén, Helene Zachrisson

**Affiliations:** 1 Department of Clinical Physiology, Linköping University, Linköping, Sweden; 2 Department of Health, Medicine and Caring Sciences, Linköping University, Linköping, Sweden; 3 Department of Thoracic and Vascular Surgerys, Linköping University, Linköping, Sweden

**Keywords:** chronic venous disease, ultrasound, venous hemodynamics, venous reflux, vascular imaging/diagnostics

## Abstract

Superficial venous incompetence (SVI) is the most common manifestation of chronic venous disease and represents a significant global health burden. Ultrasound has long been the diagnostic modality of choice in the evaluation of SVI, offering excellent anatomical information of venous reflux. However, the most widely used and currently recommended ultrasound parameter, reflux time, has shown a limited correlation with clinical severity and should primarily be considered a qualitative measure used to determine the presence or absence of venous reflux. In this context, quantitative assessments of venous reflux may provide a more nuanced and clinically informative evaluation of the severity and character of SVI. This review aims to summarize and critically assess the current evidence, as well as existing knowledge gaps, concerning quantitative ultrasound-based evaluation of SVI.

## Introduction

Chronic venous disease (CVD) arises from ambulatory venous hypertension caused by reflux, obstruction, or impaired calf muscle pump function, leading to microvascular inflammation and tissue remodeling.^1,2^ Superficial venous incompetence (SVI) is the most common manifestation of CVD, affecting 30–40% of the population and caused by valvular incompetence in the superficial venous system.^2–4^ Management strategies for SVI depend on clinical presentation; however, color duplex ultrasound (CDU) is considered mandatory in the evaluation because clinical examination alone cannot accurately identify the source or extent of venous reflux. CDU provides essential anatomical information by mapping the affected venous segments. These insights are critical for guiding appropriate management strategies, including selection and planning of endovenous or surgical interventions.^2,5–8^ Reflux time (RT) is the most used parameter as it provides a threshold that defines pathology,^9–11^ and the cut-off value most used is 0.5 seconds for superficial veins.^2^ However, RT has demonstrated a weak correlation with clinical signs and symptoms, and should be regarded primarily as a qualitative parameter, intended to determine the presence or absence of reflux.^2,12–14^

Although clinical improvement remains the goal of intervention, hemodynamic correction reflecting the normalization of flow direction is a key mechanism underlying symptom relief. Quantitative CDU parameters may assist in characterizing these underlying physiologic changes. Moreover, lower-limb symptoms may originate from various causes and are not always venous in origin. Even when venous pathology is present, SVI may not be the primary source of symptoms.^15–17^ For instance, symptoms may arise from cardiac issues, arterial disease, deep venous incompetence, or outflow obstruction. In such cases, quantitative hemodynamic assessment of the magnitude, volume, and duration of retrograde flow can serve as an objective reference, helping to differentiate of venous from nonvenous etiologies, and to clarify the specific contribution of SVI to the patient’s symptoms.

This review summarizes and critically evaluates current evidence and gaps regarding quantitative ultrasound-based assessment of SVI. Relevant literature was identified through a nonsystematic search of PubMed, Scopus, and Google Scholar using keywords such as “superficial venous incompetence,” “venous reflux,” “quantitative ultrasound,” “reflux volume,” “reflux volume flow,” “venous recirculation index,” “postural diameter change,” and “hemodynamics.” Additional references were identified by manually screening the bibliographies of key articles. Priority was given to peer-reviewed studies published in English within the last 20 years, with the literature search completed in June 2025. Studies focused on novel or clinically relevant quantitative parameters were emphasized. No formal inclusion or exclusion criteria were applied, as the goal was to provide a broad, integrated overview rather than a systematic synthesis.

## Classification, clinical scoring systems, and patient-related outcome measures

Several systems are used to classify and assess CVD, including the Clinical, Etiological, Anatomical, Pathophysiological (CEAP) classification,^18^ the revised Venous Clinical Severity Score (r-VCSS),^19,20^ and patient-reported outcome measures such as the Aberdeen Varicose Vein Questionnaire (AVVQ) and Venous Insufficiency Epidemiologic and Economic Study–Quality of Life/Symptoms (VEINES-QOL/Sym).^21,22^ These tools provide information on disease severity, clinical progression, and quality of life. A concise overview of their key features and applications is summarized in Table 1.

**Table 1.** Overview of commonly used clinical classification systems and patient-reported outcome measures in chronic venous disease.

Instrument	Purpose	Strengths	Limitations
Clinical, Etiological, Anatomical, Pathophysiological classification (CEAP)	Description of CVD across four domains. The Clinical (C) class categorizes disease severity from C0–C6.	Standardized and internationally adopted. Facilitates uniform reporting and comparison between studies.	Discriminative instrument. Not constructed for monitoring treatment response or disease progression.
Revised Venous Clinical Severity Score (r-VCSS)	Quantifies clinical disease severity and treatment-related changes across 10 items.	Evaluative instrument. Designed to measure changes in status after venous intervention.	Includes nonspecific symptoms and misses key signs (e.g., venous claudication). May over- or underestimate disease severity.
Aberdeen Varicose Vein Questionnaire (AVVQ)	Disease-specific HRQoL tool to assess symptoms, cosmetic concerns, and functional limitations in patients with varicose veins.	Validated. Responsive to treatment-related improvement. Simple to administer.	Designed for varicose veins only. Some symptoms are nonspecific and influenced by other venous or arterial diseases, reducing accuracy.
Venous Insufficiency Epidemiologic and Economic Study–Quality of Life/Symptoms (VEINES-QOL/Sym)	Venous-specific HRQoL tool to assess symptoms, function, and psychosocial impact across the full spectrum of venous disease.	Validated. Applicable to both superficial, deep, and obstructive venous disease.	Less sensitive to postintervention changes and not specific for varicose vein disease.

HRQoL, Health-Related Quality of Life.

## Quantification of venous reflux using color duplex ultrasound (CDU)

RT is defined as the duration of reflux (retrograde flow) in response to distal augmentation or a Valsalva maneuver, with a pathological threshold of ⩾ 0.5 seconds for superficial veins. Despite being the most commonly used parameter, RT correlates poorly with clinical signs and symptoms and should therefore primarily be used qualitatively to determine the presence or absence of reflux.^2,9–14^ Over the years, several hemodynamic parameters assessed by CDU have been investigated to quantify venous reflux. CDU should generally be performed with the patient in an upright position, either standing or positioned on an examination table or chair that allows for patient tilting.^23,24^ For all assessments, measurements are most commonly obtained in the great saphenous vein (GSV) at the proximal thigh. However, certain assessments, such as postural diameter change (PDC), also require measurements in the supine position. To induce venous reflux, antegrade blood flow must first be generated, which is typically achieved by compression applied below the measurement site, using either an automatic pneumatic cuff or manual compression. The additional time required to obtain these measurements is minimal, usually only a few minutes longer than standard reflux time assessment, making them feasible for integration into routine examinations. This review focuses on the parameters that have attracted the most attention and are emphasized in recent guidelines.^2,7^

### Reflux volume flow

**Figure 1.**
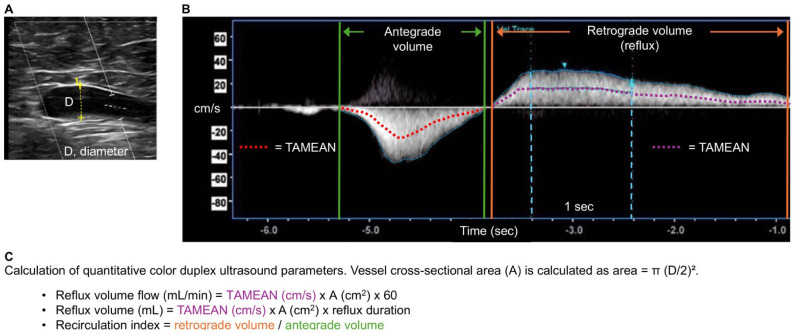
Quantitative assessment of venous reflux using color duplex ultrasound (CDU). **(A)** B-mode ultrasound image showing measurement of vessel diameter. **(B)** Example of a pulsed Doppler ultrasound measurement in the proximal great saphenous vein. An automatic compression cuff is inflated to 100 mmHg, resulting in an antegrade flow signal. Upon cuff deflation, a larger retrograde flow (reflux) is observed. Time-averaged mean velocity (TAMEAN) is indicated for both antegrade and retrograde flow phases. **(C)** Formulas used to calculate quantitative CDU parameters. By measuring the vessel diameter in conjunction with Doppler parameters, reflux volume flow, reflux volume, and the recirculation index can be calculated. Reflux volume flow is calculated using TAMEAN measured over 1 second during the period of highest flow following distal compression release. In contrast, reflux volume is calculated using TAMEAN measured over the entire reflux duration. The recirculation index expresses the ratio of retrograde to antegrade volume.

Reflux volume flow refers to the amount (volume) of blood that is refluxing within a given time. It is typically measured in milliliters per minute (mL/min) and has been suggested to provide a quantitative measure of SVI.^12,25,26^ Reflux volume flow is derived from the time-averaged mean velocity (TAMEAN, cm/s), measured over 1 second during the period with the highest flow rate after release of distal compression, and the cross-sectional area of the vessel, which is considered to be circular (*A* = πr^2^). Thus, reflux volume flow (mL/min) is calculated as: reflux volume flow (mL/min) = TAMEAN (cm/s) × *A* (cm^2^) × 60 (Figure 1A–C).

Reflux volume flow correlates more closely with clinical severity (C in CEAP) than reflux time, with higher reflux volume flow in patients with C4–C6 disease compared to C2–C3 disease.^13,14,26^ Despite its promise, reflux volume flow is subject to several limitations. Proposed thresholds for classifying reflux severity remain unvalidated,^27^ and no universally accepted reference values exist, limiting its routine use. Moreover, as reflux volume flow is calculated over a fixed time window, it may not capture the full spectral profile of retrograde flow. Patients with similar reflux volume flow values can exhibit markedly different waveform morphologies, reducing its diagnostic precision.

### Reflux volume

Reflux volume refers to the total amount (volume) of blood that flows backward through the veins due to malfunctioning valves.^12,28^ It is typically measured in milliliters (mL). Reflux volume is derived from TAMEAN, the cross-sectional area of the vein, which is considered to be circular, and the duration of reflux. Thus, reflux volume is calculated as: reflux volume (mL) = TAMEAN (cm/s) × *A* (cm^2^) × reflux duration (s) (Figure 1A–C).

Reflux volume provides an alternative to reflux volume flow, quantifying the total volume of retrograde blood and reflecting the overall hemodynamic burden. Higher reflux volumes have been observed in patients with more advanced clinical manifestations, such as skin changes and ulceration, compared to those without cutaneous involvement.^28,29^ However, current evidence does not show superior diagnostic or discriminatory value over reflux volume flow, and standardized reference thresholds are lacking.^12,28^ Moreover, reflux volume largely depends on the ejected antegrade volume during compression, suggesting that differences may reflect technical aspects of the diagnostic procedure rather than the true pathophysiological burden.^29^

### Recirculation index

Because the antegrade volume, specifically the ejection volume generated by the reflux-provoking maneuver, is a primary determinant of reflux volume, the recirculation index (RCI) has been proposed as a more standardized metric to quantify reflux.^29,30^ This is because the index normalizes the retrograde volume relative to the antegrade volume. Both antegrade and retrograde volumes are calculated using the same methodology as described for reflux volume. The RCI is then determined using the formula: RCI = reflux volume / antegrade volume. It is assumed that the diameter of the GSV remains constant during both reflux and antegrade flow, leading to its cancellation in the equation (Figure 1A–C). Recirculation is typically defined as RCI > 1, indicating that the reflux volume exceeds the preceding antegrade volume.^30^

The concept of recirculation has long been recognized in venous physiology but was more recently reintroduced by Lattimer et al. in the context of SVI.^30^ In a comparison of three ultrasound-derived parameters and the venous filling index (VFI) obtained from air plethysmography, the RCI demonstrated the highest discriminative value for identifying significant reflux among all ultrasound-based measures.^16^ However, only a limited number of studies have investigated RCI, all of which have involved relatively small sample sizes.^16,30,31^ Consequently, no comprehensive evaluation has been conducted to assess the correlation between RCI and disease severity.

### Postural diameter change (PDC)

**Figure 2.**
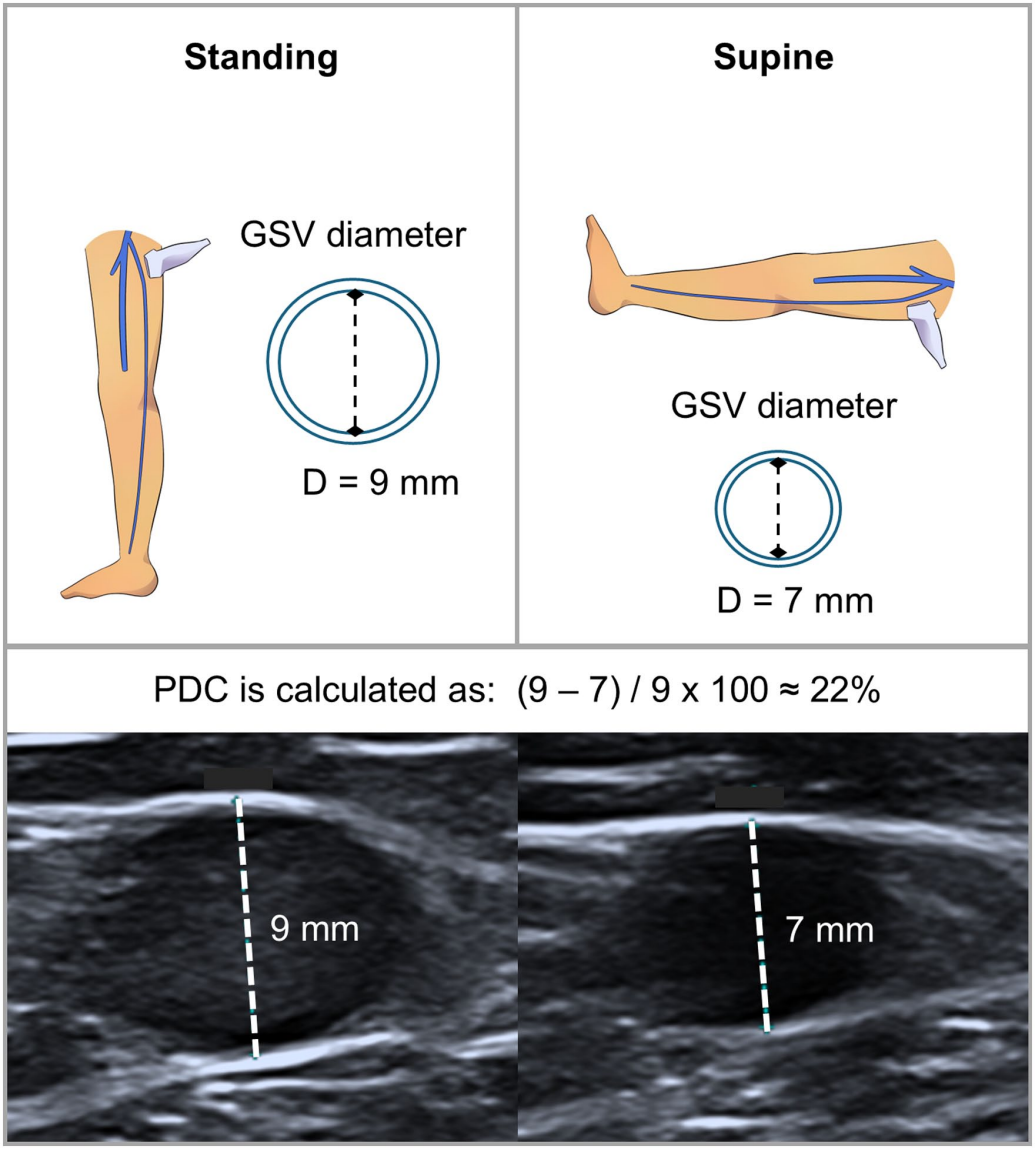
Measurement of postural diameter change (PDC) of the great saphenous vein (GSV) based on the anteroposterior inner vein diameter. The diameter measured in the standing position is compared with that measured in the supine position. In this example, the PDC is 22%.

PDC is defined as the percentage reduction in the inner diameter of the examined vessel when transitioning from a standing to a supine position.^32^ This measurement is based on the premise that varicose veins exhibit altered vessel wall properties, leading to impaired venous wall elasticity.^33–35^ Specifically, in cases of reduced vein wall elasticity, the decrease in diameter during the postural transition is expected to be less pronounced compared to that observed in a healthy limb.^32^ The formula for calculating PDC is as follows: PDC (%) = standing diameter (mm) – supine diameter (mm) / standing diameter (mm) × 100 (Figure 2).

PDC has been shown to be significantly reduced in patients with varicose veins compared to individuals with healthy veins, and further diminished in those with C4–C6 disease relative to those with C2–C3 disease.^16,31,32^ However, PDC is highly operator-dependent and influenced by transducer pressure, ambient temperature, and the patient’s hydration status, all of which may contribute to inter- and intra-observer variability and thereby compromise the reproducibility of the assessment. Moreover, no standardized reference values or pathological thresholds have yet been established.

## Quantification of global venous function using plethysmography

**Figure 3.**
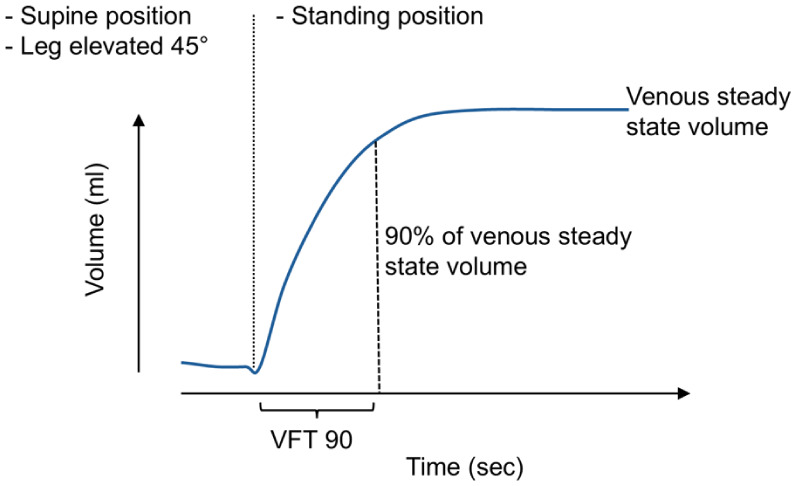
A typical air plethysmographic recording showing changes in leg volume during postural transition. As the subject moves from the supine position with the leg elevated at 45° to a standing position, an increase in leg volume is observed due to venous filling. The time required to reach 90% of the total venous volume is defined as the venous filling time 90 (VFT 90). The venous filling index (VFI, mL/s) is calculated as 90% of the venous volume divided by VFT 90.

**Figure 4.**
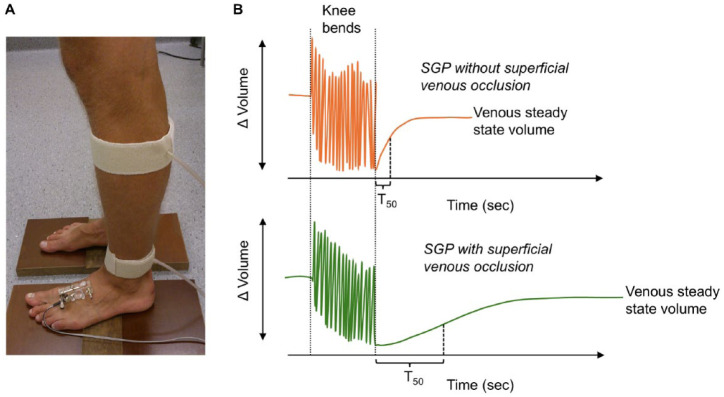
**(A)** Example of the methodological setup for strain-gauge plethysmography using standardized superficial venous occlusion. A calf cuff, ankle cuff, and strain gauge are applied for the measurement of volume changes in the foot. **(B)** A typical strain-gauge plethysmography (SGP) recording during a series of knee bends followed by quiet standing, shown without (top) and with (bottom) superficial venous occlusion in a patient with great saphenous vein incompetence. The time to reach 50% of the total venous volume (T_50_) is markedly shorter in the absence of occlusion, indicating significant reflux within the superficial venous system.

Although this review primarily focuses on ultrasound, plethysmography is briefly included as a complementary technique for quantifying SVI. Air plethysmography measures limb volume using an air-filled cuff during postural change (Figure 3), whereas distal strain-gauge plethysmography (SGP) records foot volume changes using a strain gauge with and without standardized superficial venous occlusion during a series of knee bends (Figures 4A and 4B). Thus, plethysmography noninvasively measures limb volume changes, reflecting global venous hemodynamics rather than isolated vein segments.^36–38^ The VFI, derived from air plethysmography, is the most established parameter.^12,14,16,39^ VFI represents the rate at which venous blood refills the lower limb as a result of reflux. Elevated VFI values have been associated with higher CEAP clinical classes and VCSS, although notable individual variability and overlap have been observed.^16,39^ Plethysmographic assessment using cuff occlusion techniques has also been employed to differentiate superficial from deep venous incompetence. Distal SGP with selective superficial venous occlusion has been shown to enable the separation of superficial and deep venous refilling components.^40^ In a study by Nelzén et al., preoperative SGP reflux parameters predicted outcomes after radiofrequency ablation, suggesting utility in identifying patients most likely to benefit from intervention.^40^ Such predictive capacity may be valuable in evaluating the likelihood of a favorable clinical response to intervention, particularly in patients with combined superficial and deep venous incompetence or partial outflow obstruction. Moreover, this approach may help determine the clinical relevance of specific reflux pathways identified through duplex ultrasound. Plethysmographic studies have further revealed that many patients exhibit persistent reflux even after technically successful treatment of superficial venous incompetence.^41,42^ These patients tend to report poorer outcomes, as reflected by higher VCSS and AVVQ scores. Despite these advantages, plethysmographic methods are not widely available and are typically confined to specialized vascular centers. The described ultrasound- and plethysmography-derived parameters used to quantify venous reflux are summarized in Table 2.

**Table 2.** Summary of ultrasound and plethysmography parameters used for assessment of superficial venous incompetence.

Parameter	Definition/formula	Units	Strengths	Limitations	Clinical notes
*Ultrasound*					
Reflux time	Duration of reflux in response to augmentation or Valsalva maneuver	Seconds	Simple. Defined cut of values of 0.5 s for SVI. Recommended in guidelines.	Qualitative. Weak correlation with symptoms.	Used in clinical practice.
Reflux volume flow	TAMEAN (cm/s) × area (cm^2^) × 60	mL/min	Quantifies reflux volume flow. Correlates more closely with CEAP C-class than RT.	No validated cut-offs. Affected by Doppler waveform variation and compression technique.	May indicate disease severity. Not used in routine clinical practice.
Reflux volume	TAMEAN (cm/s) × area (cm^2^) × duration of reflux	mL	Quantifies total reflux volume. Correlates more closely with CEAP C-class than RT.	No validated cut-offs. Affected by compression technique.	May indicate disease severity. Not used in routine clinical practice.
Recirculation index	Reflux volume / antegrade volume	Ratio	Normalizes reflux to antegrade flow. RCI > 1 indicates significant reflux.	Limited studies and small sample sizes. Lacks validation against disease severity.	Promising but requires validation in larger studies. Not used in routine clinical practice.
Postural diameter change	Standing diameter (mm) – supine diameter (mm) / standing diameter (mm) × 100	%	Reflects venous wall elasticity. Correlates more closely with CEAP C-class than RT.	No validated cut-offs. Operator-dependent and variable.	May indicate disease severity. Not used in routine clinical practice.
*Plethysmography*					
Venous filling index, air plethysmography	90% Venous volume (mL) / venous filling time (s)	mL/s	Quantifies global venous reflux. Correlates with CEAP C-class and r-VCSS. The most established plethysmographic parameter.	Limited availability. Overlap between disease severities.	Not used in routine clinical practice. Restricted to specialized vascular centers.
T_50_, Distal strain-gauge plethysmography	Time to refill 50% of venous volume	Seconds	Quantifies global venous reflux. Correlates with CEAP C-class and r-VCSS. Separates superficial and deep reflux using selective superficial occlusion.	Limited availability. Overlap between disease severities.	Not used in routine clinical practice. Restricted to specialized vascular centers.

CEAP, Clinical, Etiological, Anatomical, Pathophysiological classification; PDC, postural diameter change; RCI, recirculation index; RT, reflux time; r-VCSS, revised Venous Clinical Severity Score; SVI, superficial venous incompetence; TAMEAN, time-averaged mean velocity; VFI, venous filling index.

## Discussion

Traditional qualitative parameters, such as a reflux time cut-off of > 0.5 seconds, primarily serve to confirm the presence or absence of reflux.^2^ However, reliance on reflux time alone does not adequately capture the underlying hemodynamic disturbances. Quantitative assessments may offer a more nuanced characterization of reflux severity and its physiologic consequence. Reflux volume flow, reflux volume, RCI, and PDC provide complementary measures of venous reflux. Each has shown associations with clinical severity, but none have established reference thresholds or standardized protocols, limiting their current applicability in clinical practice.

### Barriers to clinical implementation and validation needs

#### Pre- and postoperative measurements

An important consideration is whether CDU parameters can be used to evaluate treatment response and follow-up. After removal of the GSV, most parameters are no longer applicable. In cases of incomplete ablation, reflux volume flow, reflux volume, and RCI may still be assessed, whereas PDC is unsuitable due to posttreatment alterations in the venous wall structure.^16^

#### Role of compression

The volume of retrograde flow is inherently dependent on the preceding antegrade volume.^29,30^ Reflux assessments is often performed using manual compression, which introduces variability due to inconsistencies in both the applied pressure and the timing of compression and release. In a study by Lattimer et al., automated compression produced significantly higher and more consistent antegrade and retrograde volumes compared with manual methods, with Doppler velocity profiles varying markedly according to operator technique.^30^ Similarly, reflux volumes increase proportionally with inflation pressure when using an automatic compression device, until reaching a plateau at approximately 100 mmHg.^29^ These findings highlight the need for standardized compression protocols, as measured volumes may otherwise reflect technical rather than pathophysiologic differences.

#### Patients with combined superficial and deep venous incompetence

In patients with both superficial and deep venous reflux, superficial reflux volumes may be reduced due to early venous filling from deep reflux. Consequently, volumetric measurements may underestimate superficial disease and may not reliably predict the symptomatic benefit of superficial interventions.^28^

#### Isolated venous function versus global venous function

Ultrasound effectively maps the anatomical distribution of venous reflux but assesses veins in isolation rather than capturing overall venous function. Consequently, it may not fully reflect global hemodynamics. Complementary techniques, such as plethysmography (discussed above), can provide a broader assessment if needed.

#### Validation and standardization challenges

Evidence for quantitative CDU parameters remains limited. Well-designed prospective studies are needed to establish reproducible reference values and pathological thresholds. Robust validation will also require larger trials using standardized methods, automated compression systems, and correlation with clinical outcomes.

## Conclusions

Quantitative CDU parameters offer an expanded means of characterizing venous reflux beyond traditional reflux time. Measures such as reflux volume flow, reflux volume, RCI, and PDC show associations with disease severity and may complement current qualitative assessments. However, methodological variability, lack of standardized protocols, and absence of validated reference thresholds currently limit their clinical applicability. Standardized study designs, preferably using automated compression systems and correlation with clinical outcomes, are needed to determine their diagnostic value in superficial venous incompetence.
